# Self-Healing Property of Ultra-Thin Wearing Courses by Induction Heating

**DOI:** 10.3390/ma11081392

**Published:** 2018-08-09

**Authors:** Jiuming Wan, Yue Xiao, Wei Song, Cheng Chen, Pan Pan, Dong Zhang

**Affiliations:** 1 State Key Laboratory of Silicate Materials for Architecture, Wuhan University of Technology, Wuhan 430070, China; wanjm@whut.edu.cn (J.W.); songwei6695@whut.edu.cn (W.S.); chencc@whut.edu.cn (C.C.); pytmac@whut.edu.cn (D.Z.); 2 School of Civil Engineering and Architecture, Wuhan Institute of Technology, Wuhan 430205, China; panpan8597@126.com

**Keywords:** ultra-thin wearing course, self-healing, induction heating, steel fiber, steel slag

## Abstract

Ultra-thin wearing course (UTWC) has been developed in pavement preventive maintenance for many years. However, how to prolong the service life of UTWC still requires further research. This study introduced AC-5 and SMA-5 asphalt mixtures, which can be induction heated. Steel fiber and steel slag were used in the mixtures as additives. Marshall Stability and induction heating property of mixtures were characterized. In addition, self-healing property of UTWC materials had been emphatically conducted. Adding steel fiber in mixtures led to higher Marshall Stability and lower flow value, while steel slag generally showed a negative effect. Induction heating property showed a positive relationship with the additives. Induction heating time was positively correlated to the healing ratio of the mixtures. Induction heating on the mixtures could recover the strength of mixtures to a certain degree. Mixtures with more steel fiber showed a higher healing ratio. Basalt-steel slag based mixtures showed better healing ratios than the basalt based mixtures. The healing ratios of mixtures illustrated a decreasing tendency as the healing cycle increased.

## 1. Introduction

China has achieved a sharp development of civil highway transportation in the last decades, showing a positive influence on the national economy. However, pavements gradually fail due to long-time service, which reduces the traffic efficiency and driving safety. Therefore, prolonging the service life of pavement with adequate maintenance is urgently required. Preventive maintenance [1,2] is a strategy that conducting maintenance on pavement that has not been seriously damaged. It has been proven to effectively prolong the service life of pavement. In addition, preventive maintenance is more economical than reconstructing a new pavement. Paving ultra-thin wearing course (UTWC) [3,4] on pavement has been served as an effective approach for prolong the service life of pavement since many year ago. UTWC was initially developed in France [5]. It is asphalt mixture course whose thickness is between 15 and 25 mm. UTWC is usually paved on the top of surface course, performing as a new wearing course. Researchers had developed many materials for UTWC. Their results [6,7] and applications [8] proved that paving UTWC instead of reconstructing the entire pavement is both environmentally [9] and economically viable. The skid resistance [10] of the pavement can be also recovered. It has advantages of shorter construction time, fewer costs, and less resources consumption. However, how to prolong the service life of UTWC still requires further research.

Besides, recycling solid waste, such as steel slag [11], could also ease some environmental pressure. It has been applied to replace part of natural aggregate in mixture, which will reduce the excessive exploitation of natural minerals. Previous studies [1,12,13] claimed that steel-slag based asphalt concrete showed acceptable performance. It proved that using steel slag to fabricate asphalt mixture was feasible. Thereby, this study also employed steel slag in UTWC to contribute to environmental protection.

On the other hand, induction heating is conducted on asphalt mixtures to melt ice and snow [14,15]. Some researchers [16,17] heal the pavement distress and prolong the service life of pavement through induction heating. Asphalt will show a great mobility when it is heated to a certain temperature since it is a viscoelastic material. The flow of asphalt binder into the crack within the asphalt mixture could help to achieve closure of the crack [18]. Thus, the cracks can be healed and the strength of mixture is recovered to a certain degree. Therefore, materials of UTWC with self-healing function were originally introduced in this study. Conducting self-healing on the asphalt pavement could effectively prolong the service life of UTWC. It could reduce both the cost and the consumption of natural resource. AC-5 and SMA-5 asphalt mixtures that can be induction heated were introduced. They were designed to be the materials of UTWC, which could be induction heated and therefore heal the crack. Steel fiber and steel slag had been used in the asphalt mixture as the heat source. Self-healing property of the UTWC materials through induction heating was emphatically investigated. In addition, the angularity and Form Two-Dimensional (2D) of aggregates, Marshall Stability, and induction heating property of the asphalt mixtures were also characterized. This study proposed the UTWC materials with self-healing function and provide the laboratory assessment was conducted. It could help to realize the self-healing of UTWC, and therefore contribute to the environmental pressure of the maintenance engineering.

## 2. Materials

Basalt and limestone filler were used as aggregate and filler in mixtures. Basalt was prepared in Hubei Province and filler was produced in Henan Province. Steel slag that was produced in Jiangxi Province were screened to the size of 2.36–4.75 mm in order to replace the basalt of same size with same volume. Table 1 presents the properties of aggregates and filler. A high viscosity asphalt binder was employed in the mixtures. Penetration of the high viscosity asphalt at 25 °C was 4.88 mm. Softening point was 86.9 °C and ductility at 5 °C was 720 mm.

**Figure 1:**
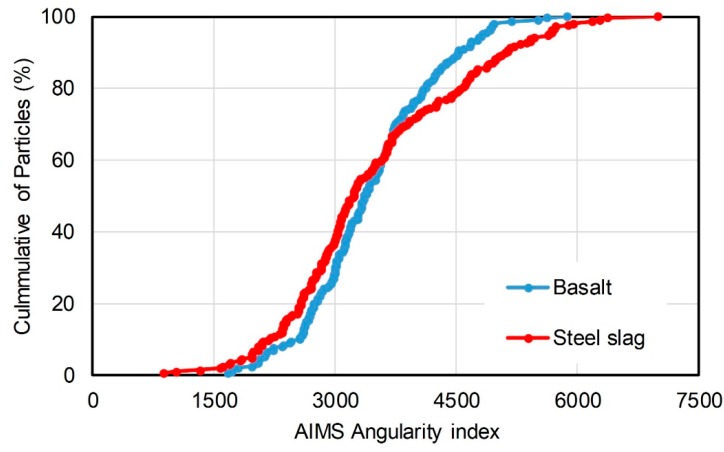
Gradient angularity of basalt and steel slag.

**Figure 2:**
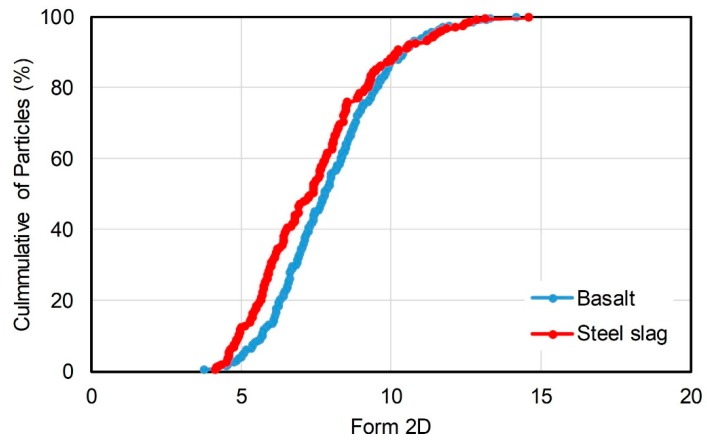
Form2D of basalt and steel slag.

Basalt and steel slag of 2.36–4.75 mm were also measured by Aggregate Image Measurement System. Cumulative distribution of corresponding angularity index and Form2D were characterized. Gradient angularity describes the variation at the particle boundary that influences the overall shape. The gradient angularity quantifies changes along a particle boundary with higher gradient values, indicating a more angular shape. Gradient angularity has a relative scale of 0 to 10,000 with a perfect circle having a small non-zero value. This index indicates the angularity, aggregates with higher angularity index prefer to show more obvious angularities. Equation (1) explains the calculation of gradient angularity:

(1) $$\mathrm{GA}=\frac{1}{\left(\frac{n}{3}-1\right)}\overset{n-3}{\underset{i=1}{\sum }}\left|\theta_{i}-\theta_{i+3}\right|$$

where GA is the gradient angularity (Dimensionless), θ is the angle of orientation of the edge points, n is the total number of points, and subscript *i* denoting the *i*th point on the edge of the particle. Form2D (Dimensionless) applies to fine aggregate sizes only and quantifies the relative form from two-dimensional images of aggregate particles. It has a relative scale of 0 to 20. Form2D indicates the sphericity of the aggregate. Aggregates of higher Form2D value show more obvious circle shape, and a perfect circular aggregate has a Form2D value of zero. The form index Form2D is expressed by Equation (2).

(2) $$\mathrm{Form}2D=\overset{\theta =360-\Delta \theta }{\underset{\theta =0}{\sum }}\left[\frac{R_{\theta +\Delta \theta }-R_{\theta }}{R_{\theta }}\right]$$

where, *R_θ_* is the radius of the particle at an angle of *θ*, *∆θ* is the incremental difference in the angle. Angularity index and Form2D describe particle shape of aggregate. Figure 1 presents the gradient angularity of basalt and steel slag. The data points of steel slag were higher than that of basalt when the angularity index is below 3700. Conversely, it showed lower cumulative of particles when the angularity index is over 3700. Figure 2 expresses the Form2D of basalt and steel slag. The cumulative of particles of steel slag were higher than that of basalt when the value of Form2D is below 10. Table 2 and Table 3 express the statistical result of particle number and proportion in different angularity range and Form2D range, which quantify the data more intuitively. The proportion of steel slag with angularity range of 5400–10,000 and 3750–10,000 were 7.39% and 29.06%, while that of basalt was 1.5% and 24%. The proportions of steel slag with high and extreme angularity were higher than basalt. According to Table 3, proportion of steel slag with Form2D range of 0–6.5 and 0–8 were 39.9% and 61.6%, while that of basalt were 23% and 55%. It suggested that proportion of steel slag with circular shape and moderate circular shape were higher than basalt. Therefore, steel slag that was used in this study had a more circular shape and higher angularity than basalt.

Steel fiber with length of 4.2–5.0 mm and equivalent diameter of 70–130 μm was used. The density and the melting point of the steel fiber were 7.85 g/cm^3^ and 1530 °C. It could help to realize the induction heating function of mixtures. Additionally, the lignin fiber was used as stabilizer in SMA-5 mixture, its length was of 4–6 mm and PH value was 7.5 ± 1.0. The oil absorption rate of the lignin fiber was more than five times of the fiber mass, and the mass ratio of lignin fiber-aggregate was 0.3%.

## 3. Research Program

### 3.1. Mixture Design

**Figure 3:**
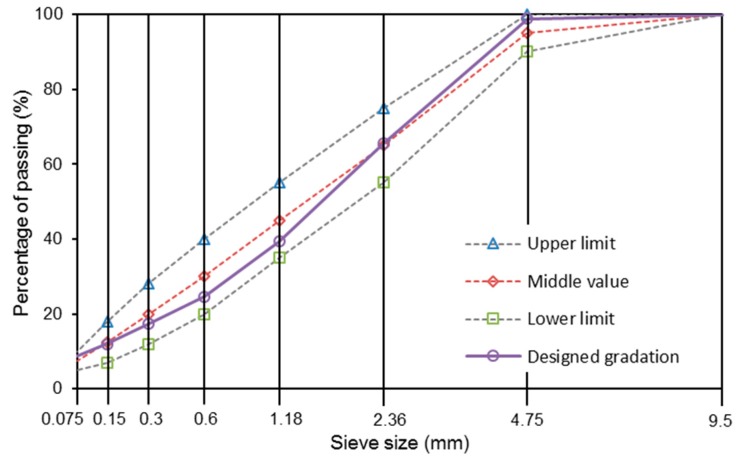
Gradations of AC-5 mixtures.

**Figure 4:**
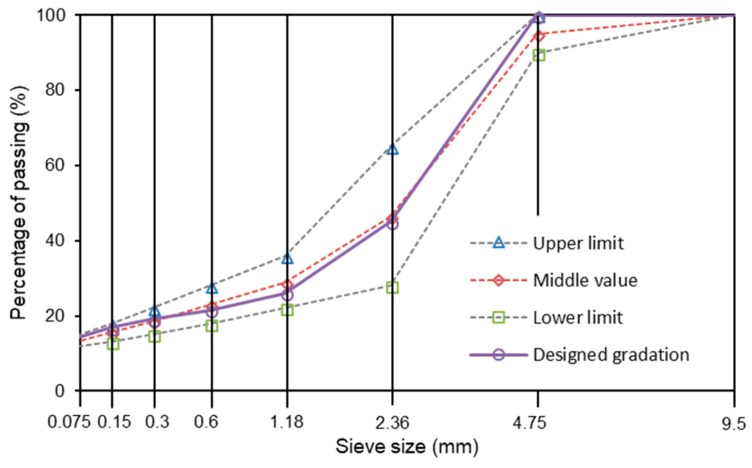
Gradations of SMA-5 mixtures.

AC-5 asphalt mixture had been designed according to the specification “JTG F40 2004”, which specifies the design method and the corresponding standards of asphalt mixture in China. SMA-5 asphalt mixture was designed according to AASHTO. Mixtures without steel slag were named as basalt based mixtures. Steel slag of 2.36–4.75 mm was proposed to replace the same size basalt as the same volume. Mixtures that contained steel slag were labelled as basalt-steel slag based mixtures. Figure 3 and Figure 4 illustrate the gradations of AC-5 and SMA-5 mixtures. The replacement of steel slag does not change the corresponding designed gradations. For all AC-5 mixtures, the binder-aggregate mass ratio was 6.2%. The asphalt binder-aggregate mass ratio of all SMA-5 mixtures was 6.5%. The mixing temperature was 175 °C, and the mold temperature was 165 °C.

On the other hand, steel fiber was added in the mixtures. Contents of steel fiber in each mixture were 0%, 1%, 2%, and 3%.

### 3.2. Testing and Characterization

Testing and characterization on materials consist of three parts, namely the Marshall Stability test, induction heating property, and self-healing property. The standard of this test is “JTG E20-2011”, which sets the standard experiment method in China. Marshall Stability test reflects the fracture strength of asphalt mixture. Four parallel Marshall specimens were tested for each mixture. These specimens were kept in a water bath with a temperature of 60 °C ± 0.5 °C for 30 min. These specimens were then set on a Marshall Stability Apparatus, and corresponding Marshall Stability and flow value of Marshall specimens were determined after testing. The loading rate for this test was 50.0 mm/min, and it was stopped when the Marshall specimens failed.

Average temperature rising rate of the materials after induction heating was investigated to indicate induction heating property. Marshall specimens were introduced for induction heating. Induction heating equipment was employed to heat the Marshall specimens. A FLIR infrared camera was used to record the infrared image of specimens after induction heating. The distance between equipment and specimens was 15 mm. The power of equipment was 7.9 kilowatts and the frequency was 124 kHz. Induction heating time was 30 s. A software named FLIR Tool was used to calculate the average temperature of the Marshall specimens. Induction heating property of the materials was therefore concluded by testing the average temperature rising rate of the mixtures during the induction heating period.

**Figure 5:**
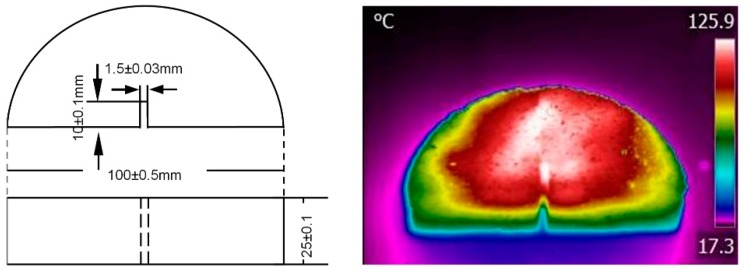
Description of the semi-circular specimen and infrared image after induction heating.

**Figure 6:**
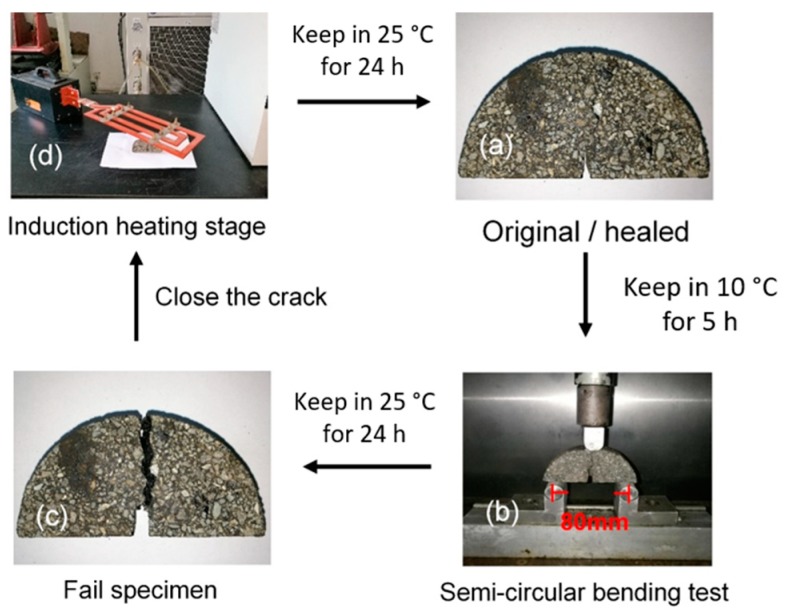
The self-healing cycle: (**a**) The original/healed specimen; (**b**) Semi-circular bending test in UTM-25; (**c**) The fail specimen; (**d**) Induction heating on specimen for healing.

The self-healing property was characterized by semi-circular bending test. Semi-circular bending test at −10 °C is a test that indicates the low temperature cracking resistance of asphalt mixture. The description of semi-circular specimens and infrared image after induction heating is shown in Figure 5. Semi-circular specimens were fabricated by cutting Marshall specimens. A notch with depth of 10 mm was made. Therefore, there would be a stress concentration along the notched part of the specimen when loading was applied. Figure 6 presents a complete cycle of self-healing analysis. Semi-circular specimens were stored at −10 °C for 5 h before test. A Universal Testing Machine (UTM-25) with an upper limit of 25 kN was used to exert loading on specimens as b stage of Figure 6. Testing temperature was −10 °C and loading rate was 0.5 mm/min. Loading stopped as force began to decrease, then the peak loading could be recorded. As shown in stage c, the broken specimens were put in a temperature control box at 25 °C for 24 h. The last stage was illustrated in part d. Pieces of specimens were put together and induction heated. Consequently, pieces of specimens could be bonded together. Thus, specimens were self-healed. After that, the healed specimens were kept in a temperature control box at 25 °C for 24 h and the self-healing circle was completed. Four self-healing circles were performed on each specimen to investigate the persistence of self-healing of the asphalt mixtures.

## 4. Result and Discussion

### 4.1. Marshall Stability and Flow Value

**Figure 7:**
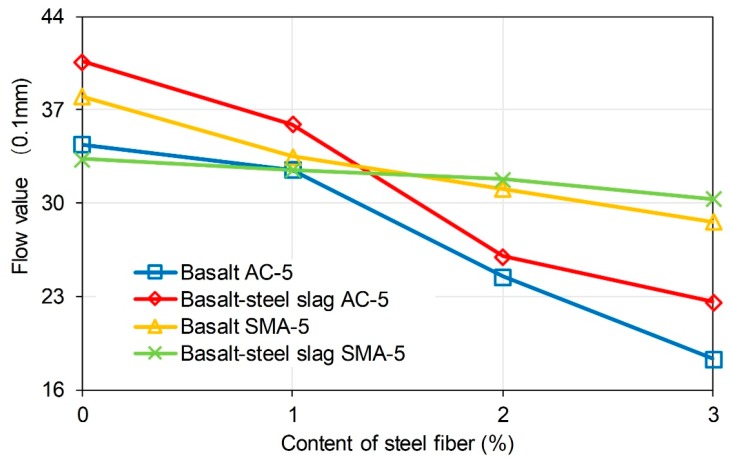
Marshall stability of the mixtures.

**Figure 8:**
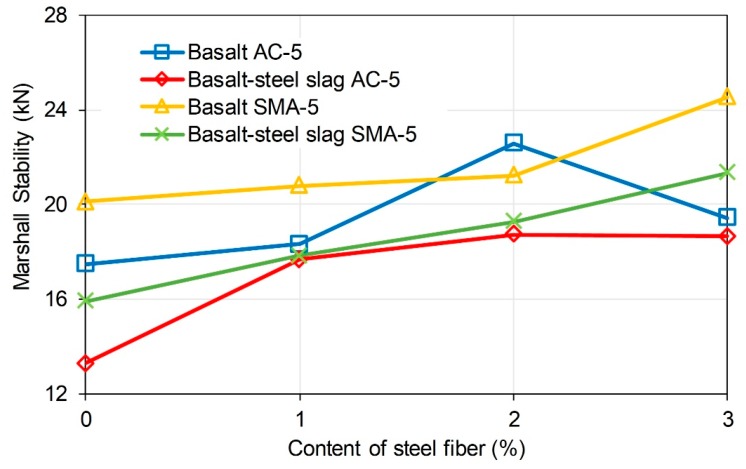
Flow value of the mixtures.

Marshall Stability illustrates the high temperature fracture strength of a sample under loading. Flow value indicates the deformation that asphalt mixture can endure when fracture happens. These indicators are used as the important properties for asphalt mixture design and testing. Asphalt mixture with inadequate Marshall Stability and flow value is thought to have poor mechanical property. Figure 7 illustrates the Marshall Stability of the mixtures. Basalt-steel slag based AC-5 and SMA-5 asphalt mixture presented an increasing tendency as the content of steel fiber in mixture increased from 0 to 3%. Basalt based mixture also showed a rising tendency when the content of steel fiber increased from 0 to 2%. However, when the content of steel fiber went up to 3%, Marshall Stability of the specimens showed a decreasing tendency. Researchers had reported that the addition of fibers would reinforce asphalt mixture. Hence, Marshall Stability rises as the content of steel fiber increases. However, excessive addition of steel fiber may harm the structure of mixture and limit the effective asphalt binder. Consequently, the fracture strength will decrease when the content of steel fiber is added. Besides, basalt-steel slag based mixture presented lower Marshall Stability than corresponding basalt based mixture. It proved that the addition of steel slag would negatively affect the fracture strength of asphalt mixture. But, the reduction resulting from steel slag was acceptable since Marshall Stability of these mixtures met the specification of 8 kN. Figure 8 shows the flow value of mixtures. Flow values of the specimens gradually decreased as the content of steel fiber raises. Addition of steel slag would raise flow value of both basalt based and basalt-steel slag based AC-5 mixtures. 

### 4.2. Induction Heating Property

UTWC that was introduced in this study is designed to have a self-heling function, so the average temperature rising rate of these materials during induction heating is important. Table 4 illustrates the average temperature rising rate of mixtures during induction heating time of 30 s. Mixtures without steel fiber showed no temperature change after induction heating. Average temperature rising rate of all the mixtures presented a rising tendency as the content of steel fiber increased. Rising rate of temperature of the basalt-steel slag based mixtures could even reach over 1 °C/s. Additionally, average temperature rising rate of basalt-steel slag based mixtures were higher than that of the corresponding basalt based mixtures. In summary, the addition of steel fiber and steel slag had a positive correlation to the induction heating property of UTWC.

### 4.3. Self-Healing Property

**Figure 9:**
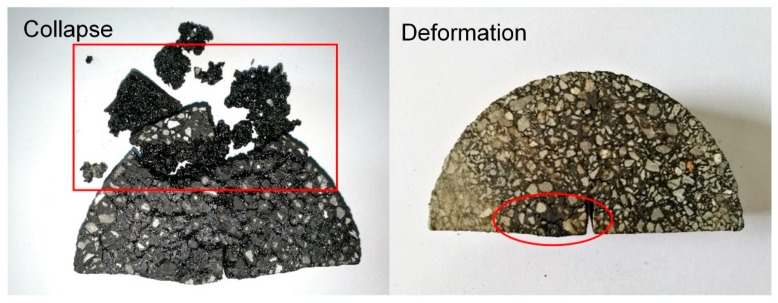
Collapse (**left**) and deformation (**right**) of the semi-circular specimens caused by excessive high temperature.

The influence of steel slag and steel fiber on self-healing property of asphalt mixtures was emphatically investigated. However, an excessive time of induction heating of specimens would excessively raise the temperature of the asphalt mixture. As Figure 9 shows, the excessive average temperature rising rate led to softening of asphalt binder, which resulted in the deformation or collapse of the asphalt mixtures. Therefore, induction heating time was controlled, and hence the average temperature rising rate of the asphalt mixture. It was noted that, the deformation of asphalt mixture initially happens when the highest temperature point of the specimen was between 120 and 130 °C. Thus, the upper limit of induction heating time of mixtures with 3%, 2%, and 1% steel fiber were controlled at 120 s, 140 s, and 200 s. Mixtures did not show deformation and collapse with these induction heating times and the corresponding cracks can be healed to a certain degree.

Gradient induction heating time and self-healing cycle were, respectively, conducted. Bending test at −10 °C was conducted on semi-circular specimens. Fracture toughness states the cracking resistance of asphalt mixtures at low temperature. Equations (3) and (4) illustrate the computation [19,20] of fracture toughness. Specimen with higher fracture toughness have better cracking resistance.

(3) $$K_{IC}=Y_{I}\sigma_{0}\sqrt{\pi a}$$

(4) $$\sigma_{0}=\frac{P_{C}}{2rt}$$

where

$K_{IC}$ = fracture toughness (MPa × m^0.5^);
$Y_{I}$ = the normalized stress intensity factor that is a constant in this study (Dimensionless);
$P_{C}$ = the critical load (N);
r = radius of specimens (m);
t = the specimen thickness (m); and,
a = the notch length (m).

**Figure 10:**
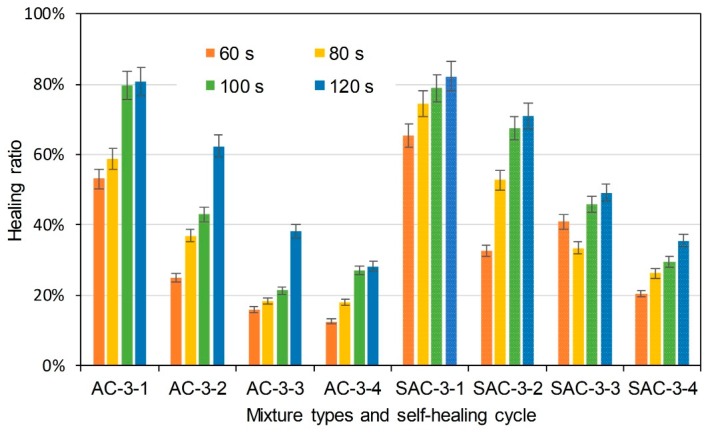
Healing ratio of basalt based and basalt-steel slag based AC-5 mixtures with 3% steel fiber by induction heating time and self-healing cycle dependency.

**Figure 11:**
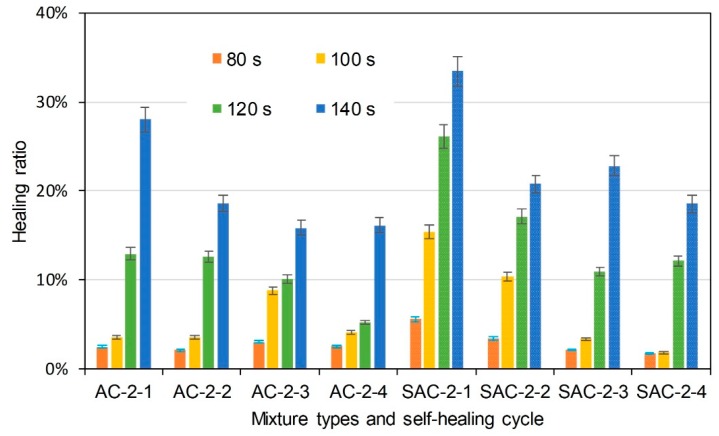
Healing ratio of basalt based and basalt-steel slag based AC-5 mixtures with 2% steel fiber by induction heating time and self-healing cycle dependency.

**Figure 12:**
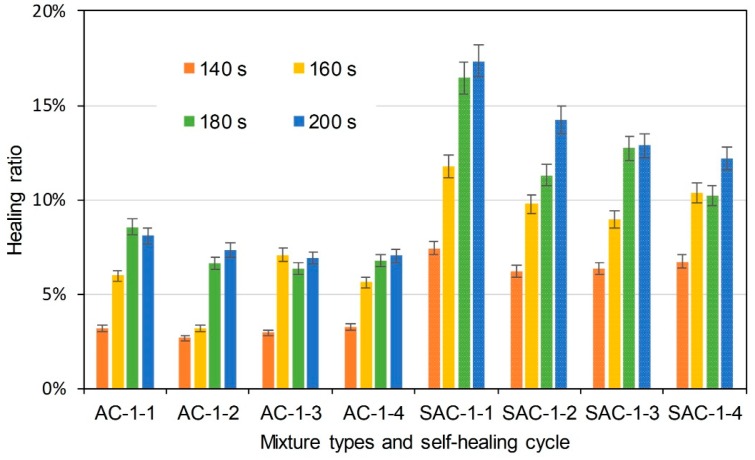
Healing ratio of basalt based and basalt-steel slag based AC-5 mixtures with 1% steel fiber by induction heating time and self-healing cycle dependency.

The same semi-circular bending tests were conducted on the initial/healed specimens after the induction heating. Healing ratio was used to indicate the self-healing property. It illustrates the degree that the specimens would recover after induction heating. It is determined according to Equation (5):

(5) $$\mathrm{HR}=\frac{K_{IC}{}_{i}}{K_{IC}}\times 100\%$$

where HR is healing ratio (%), $K_{IC}{}_{i}$ (MPa × m^0.5^) is the fracture toughness of specimens that had been healed as cycle *i*, and $K_{IC}$ (MPa × m^0.5^) is the initial fracture toughness of the specimens. Figure 10 illustrates the healing ratio of basalt based and basalt-steel slag based AC-5 mixtures with 3% steel fiber by induction heating time and self-healing cycle dependencies. AC and SAC are the abbreviations of basalt based AC-5 mixture and steel slag-basalt based AC-5 mixture. SMA and SSMA are the abbreviations of basalt based SMA-5 mixture and steel slag-basalt based SMA-5 mixture. Steel fiber content and cycle times were also included in the abbreviation of specimens. The abbreviation is introduced as the format of mixture type-steel fiber content-cycle time. For instance, AC-3-1 means the first semi-circular bending test on basalt based AC-5 specimen, which contains 3% steel fiber. SSMA-3-2 means the second semi-circular bending test on steel slag-basalt based SMA-5 mixture that contains 3% steel fiber. Bars of the left side are the healing ratio of basalt based AC-5 asphalt mixtures, and the bars with small white dots are that of basalt-steel slag based AC-5 asphalt mixtures. Different colors distinguish induction heating time on specimens from 60 to 120 s. Information of abscissa axis shows the mixture type and corresponding self-healing cycle time. Basalt-steel slag based AC-5 mixtures showed higher healing ratio at the same induction heating time and cycle. Increase of induction heating time increased the healing ratio. The healing ratio of first cycle could even reach 80%. Healing ratio of specimens showed a decreasing tendency as the cycle time increase. Figure 11 and Figure 12 present the healing ratio of AC-5 mixtures with 2% and 1% steel fiber. Basalt-steel slag based mixtures also showed higher healing ratio when compared to the corresponding basalt based mixture. Induction heating times are also correlated to the healing ratio of mixtures. Besides, the reduction of steel fiber in AC-5 asphalt mixture showed a negative effect on the healing ratio of mixture. The highest healing ratio of AC-5 mixtures with 2% and 1% steel fiber were 33.5% and 17.3%, which were far lower than that of AC-5 mixture with 3% steel fiber.

**Figure 13:**
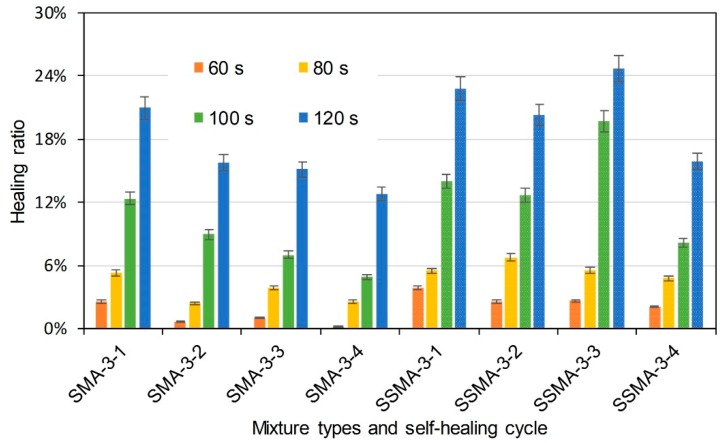
Healing ratio of basalt based and basalt-steel slag based SMA-5 mixtures with 3% steel fiber by induction heating time and self-healing cycle dependency.

**Figure 14:**
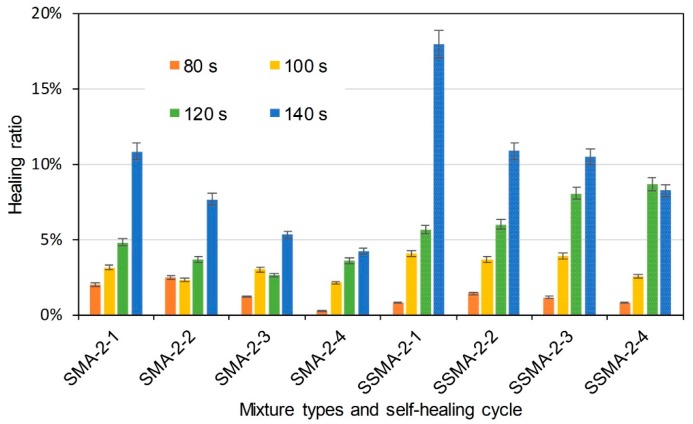
Healing ratio of basalt based and basalt-steel slag based SMA-5 mixtures with 2% steel fiber by induction heating time and self-healing cycle dependency.

**Figure 15:**
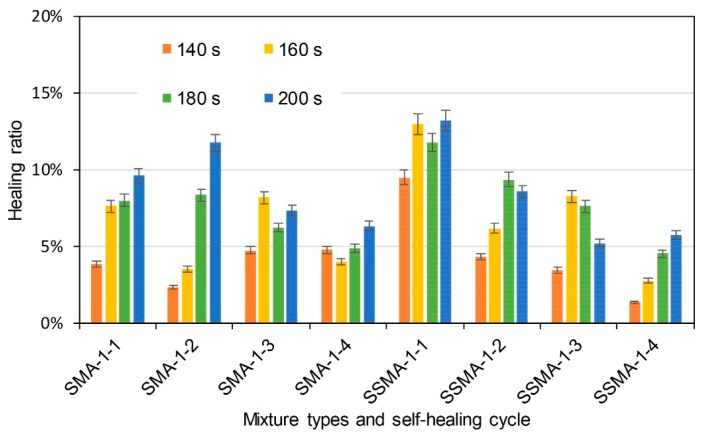
Healing ratio of basalt based and basalt-steel slag based SMA-5 mixtures with 1% steel fiber by induction heating time and self-healing cycle dependency.

Figure 13, Figure 14 and Figure 15 illustrate the healing ratio of SMA-5 mixtures. The effects of induction heating time, steel slag, and cycle were similar to that of AC-5 mixtures. Nevertheless, healing ratios of SMA-5 mixtures were very low when compared to AC-5 mixture. Healing behaviour of asphalt mixture is based on the flow of asphalt binder around crack. Asphalt binder in AC-5 mixture is supposed to easily bind the crack. However, lignin fiber in SMA-5 mixture can fix the asphalt binder, resulting in a poor mobility of the asphalt in healing time. Therefore, difference of asphalt mobility determines the healing ratio of AC-5 and SMA-5 mixtures. In summary, induction heating on the mixtures could recover the strength of the mixtures to a certain degree. Induction heating time was positively correlated to the healing ratios of mixtures. Within the adequate range, longer induction heating time will lead to better self-healing property. Basalt-steel slag based mixtures showed better healing ratios than basalt based mixtures. Mixtures with more steel fiber showed higher healing ratio in same induction heating time than mixtures with less steel fiber. It proved that the addition of steel slag could help to raise the self-healing property of mixtures. The healing ratio of mixtures illustrates a decreasing tendency as the number of cycle increase. The healing properties of SMA-5 mixtures were generally lower than that of AC-5 mixtures.

## 5. Conclusions

SMA-5 and AC-5 asphalt mixtures were designed as ultra-thin wearing course materials that could be induction heated. Steel slag, as well as steel fiber, were employed as additives in corresponding asphalt mixtures, to accelerate the induction heating speed. Marshall Stability, induction heating property, and self-healing property of the materials were characterized. The following conclusions could be drawn.

(1) Steel slag lowers slightly the Marshall Stability of asphalt mixture, while steel slag presented positive effect. Marshall Stability of basalt–steel slag based AC-5 and SMA-5 asphalt mixture presented an increasing tendency with the increased content of steel fiber from 0 to 3%. Basalt based mixture also showed a rising tendency when the content of steel fiber increase from 0 to 2%. However, when the content of steel fiber goes up to 3%, the Marshall Stability of specimens showed a decreasing tendency. Flow values of specimens gradually decreased as the content of steel fiber raises. The addition of steel slag raised the flow values of both basalt based and basalt-steel slag based AC-5 mixtures. Basalt-steel slag based mixture presented lower Marshall Stability than the corresponding basalt based mixtures.

(2) Steel slag enhances the induction heating speed. The induction heated average temperature rising rate of all mixtures presented a rising tendency as the content of steel fiber increased. Average temperature rising rate of basalt-steel slag based mixtures reached over 1 °C/s. Additionally, the average temperature rising rate of basalt-steel slag based mixtures were higher than that of corresponding basalt based mixtures.

(3) Mixtures with steel slag showed higher healing ratio than mixtures without steel slag. Adequate induction heating on mixtures could recover the strength to a certain degree. Within the adequate range, induction heating time was positively correlated to the healing ratios of mixtures. However, excessive heating time would deteriorate the mixture due to high temperature deformation. Mixtures with 3% steel fiber show a higher healing ratio within the same induction heating time than mixtures with 2% or 1% steel fiber. Basalt-steel slag based mixtures showed higher healing ratios than basalt based mixtures. 

## Figures and Tables

**Table 1:** Properties of aggregates and filler.

Properties	Apparent Specific Gravity	Bulk Specific Gravity	Water Absorption (%)	Crushing Value (%)	Content of f-CaO (%)
Basalt	2.84	2.75	1.12	12.6	-
Steel slag	3.2	2.86	2.77	14.3	1.17
Limestone filler	2.67	2.67	-	-	-

**Table 2:** Particle number and proportion in different angularity range.

Angularity Range	Basalt		Steel Slag	
	Particle Number	Proportion	Particle Number	Proportion
Low (≤2100)	9	4.50%	18	8.87%
Moderate (2100–3975)	143	71.50%	126	62.07%
High (3975–5400)	45	22.50%	44	21.67%
Extreme (5400–10,000)	3	1.50%	15	7.39%

**Table 3:** Particle number and proportion in different Form2D range.

Form2D Range	Basalt		Steel Slag	
	Particle Number	Proportion	Particle Number	Proportion
Low (≤6.5)	46	23.0%	81	39.9%
Moderate (6.5–8)	64	32.0%	44	21.7%
High (8–10.75)	75	37.5%	62	30.5%
Extreme (10.75–20)	15	7.5%	16	7.9%

**Table 4:** Average temperature rising rate of the mixtures during induction heating.

Description of Mixture		Content of Steel Fiber			
		0%	1%	2%	3%
Average temperature rising rate (°C/s)	Basalt AC-5	0	0.31	0.61	0.99
	Basalt SMA-5	0	0.34	0.59	1.02
	Basalt-steel slag AC-5	0	0.37	0.70	1.11
	Basalt-steel slag SMA-5	0	0.36	0.74	1.25
